# SWOT analysis on veterinary telemedicine from pet owner and expert perspectives—a mixed-methods survey and interview study in Germany

**DOI:** 10.3389/fvets.2026.1731577

**Published:** 2026-01-22

**Authors:** Charline Karsch, Andrea Tipold, Christin Kleinsorgen

**Affiliations:** 1 Centre for Teaching, E-Learning-Services, University of Veterinary Medicine Hannover, Hannover, Germany; 2 Clinic for Small Animals, University of Veterinary Medicine Hannover, Hannover, Germany

**Keywords:** owner perspective, small animal medicine, SWOT-analysis, telemedicine, willingness to pay

## Abstract

**Introduction:**

Telemedicine is becoming relevant for veterinarians and pet owners alike. It is insufficient to merely consider the technical requirements. Acceptance, practical applicability and actual user demand are equally important. This research study explores the potential for telemedicine services in small animal practices, examining the perspectives of pet owners and veterinary experts.

**Methods:**

A parallel mixed-method design was conducted, comprising a survey of 404 pet owners and interviews with veterinary experts. The primary objective of the study was to explore whether consumers would find an expanded range of telemedicine services appealing and to evaluate the (dis-)advantages of digital veterinary services with respect to animal welfare and owner satisfaction. Additionally, the role of digital services and a first overview of the willingness to pay for these were investigated.

**Results:**

The survey indicated that digital forms of communication are frequently utilised to contact veterinary practices. Conversely, audiovisual formats play a minor role. Veterinarians and pet-owners appreciate time savings, flexibility and stress reduction for animals as advantages. The lack of physical examination and technical hurdles are considered the main disadvantages. The willingness to pay for healthcare services is influenced by the format: video consultations are more likely to be accepted, while text-based services are often expected to be free. Expert interviews served to broaden the scope of discussion, offering insights into the practical implementation of telemedicine methodologies. SWOT analysis revealed that primary strengths of veterinary telemedicine are time savings, flexibility and stress reduction for both animals and their owners.

**Discussion:**

This digitalisation is regarded as a potential catalyst for enhancing access to and quality of veterinary care, particularly in regions characterised by structural deficiencies or for cases that necessitate specialists´ expertise. Conversely, the following limitations must be acknowledged: firstly, absence of physical examinations; secondly, presence of technical challenges; thirdly, occasional inadequacy of digital competencies. Risks associated with telemedicine include legal uncertainties, data protection concerns and the potential weakening of the personal veterinarian-client relationship due to external providers. Achieving sustainable integration necessitates the establishment of clear legal requirements, technical standards and educational initiatives. These measures are crucial for fostering trust and ensuring the effective incorporation of telemedicine into routine clinical practice.

## 1 Introduction

Due to advances in digitalisation, veterinary care is undergoing fundamental changes. Many pet owners already use digital communication channels with their veterinarians for purposes such as transmitting images of injuries, discussing lab results over the phone, or clarifying medication questions via chat, often without being aware that these activities constitute telemedicine services (1). According to the German Federal Chamber of Veterinarians (BTK), telemedicine is defined as a collective term for veterinary care concepts in diagnostics, therapy, prophylaxis, and consultation across geographical distances using digital media (2).

The acceptance of digital technologies in healthcare is frequently explained using theoretical frameworks such as the Technology Acceptance Model (TAM) (3) and the Unified Theory of Acceptance and Use of Technology (UTAUT) (4). These models emphasise that perceived usefulness, perceived ease of use, social influence and facilitating conditions are central determinants shaping individuals’ willingness to adopt new technologies. In human telemedicine research, TAM and UTAUT have repeatedly demonstrated that factors such as trust in providers, expected convenience and confidence in handling digital tools strongly influence users’ behavioural intentions (5). Preliminary findings suggest a surge in interest, particularly in structurally weak or rural regions where deficits in veterinary care exist (6). In the United States, the proportion of video consultations in small animal practices increased from 4.2 to 29.6% during the COVID-19 pandemic, particularly for behavioural counselling, dermatology, and follow-up care (7). Similarly, in Germany, COVID-19 accelerated telemedicine adoption (8). Kastelic and Ogilvie (9) predict dynamic further development, driven by technological innovation and growing social acceptance. The increasing integration of artificial intelligence (AI), such as automated radiograph analysis, intelligent blood and faecal testing devices (10) and digital monitoring tools (11), illustrate this trajectory.

To address these developments legally and organisationally, the BTK issued initial recommendations in 2020 and telemedicine guidelines in 2022 (2). These provide a framework but still impose restrictions: only licensed veterinarians may provide telemedical services, diagnoses are provisional, prescriptions are limited, billing follows the German Veterinary Fee Schedule (GOT) and advertising for prescription drugs is prohibited. In comparison, remote treatment in human medicine is more extensively regulated and allows prescriptions, sick notes, and referrals without prior in-person contact, if medically justified (12). In veterinary medicine, however, diagnostic and therapeutic decisions depend on owners acting as intermediaries, whose symptom reports may not always match clinical observations (13). International research indicates substantial variation in telemedicine adoption: countries such as Denmark, Sweden, and Estonia demonstrate markedly higher utilisation rates, supported by advanced digital infrastructure, early political commitment and user-centred e-health strategies (14). In the United Kingdom and the United States, commercial veterinary telemedicine platforms emerged earlier due to clearer regulatory systems and higher consumer digital literacy (15, 16). Insights from human telemedicine also highlight demographic moderators such as age, digital affinity, previous telemedicine experience and perceived trustworthiness of digital health services (5), which are likely transferable to pet owners. Telemedicine has been shown to provide practical benefits in Germany, particularly in underserved regions or emergency situations (17). Moreover, evolving social dynamics, including the human-animal bond shifting towards pets as family members (18), and generational changes in digital preferences, drive expectations for fast, convenient, and location-independent veterinary services (19). Against this background, the aim of this research is to assess the perspectives and expectations of pet owners regarding telemedicine in small animal practice. The study focuses on acceptance, perceived usefulness, preliminary exploration regarding the willingness to pay, and potential challenges of telemedicine. Using a mixed-method approach that integrates quantitative surveys of pet owners and qualitative interviews with experts from the veterinary field, this study seeks to develop a comprehensive understanding of the opportunities, limitations and future potential of digital veterinary services.

## 2 Methods

**Figure 1:**
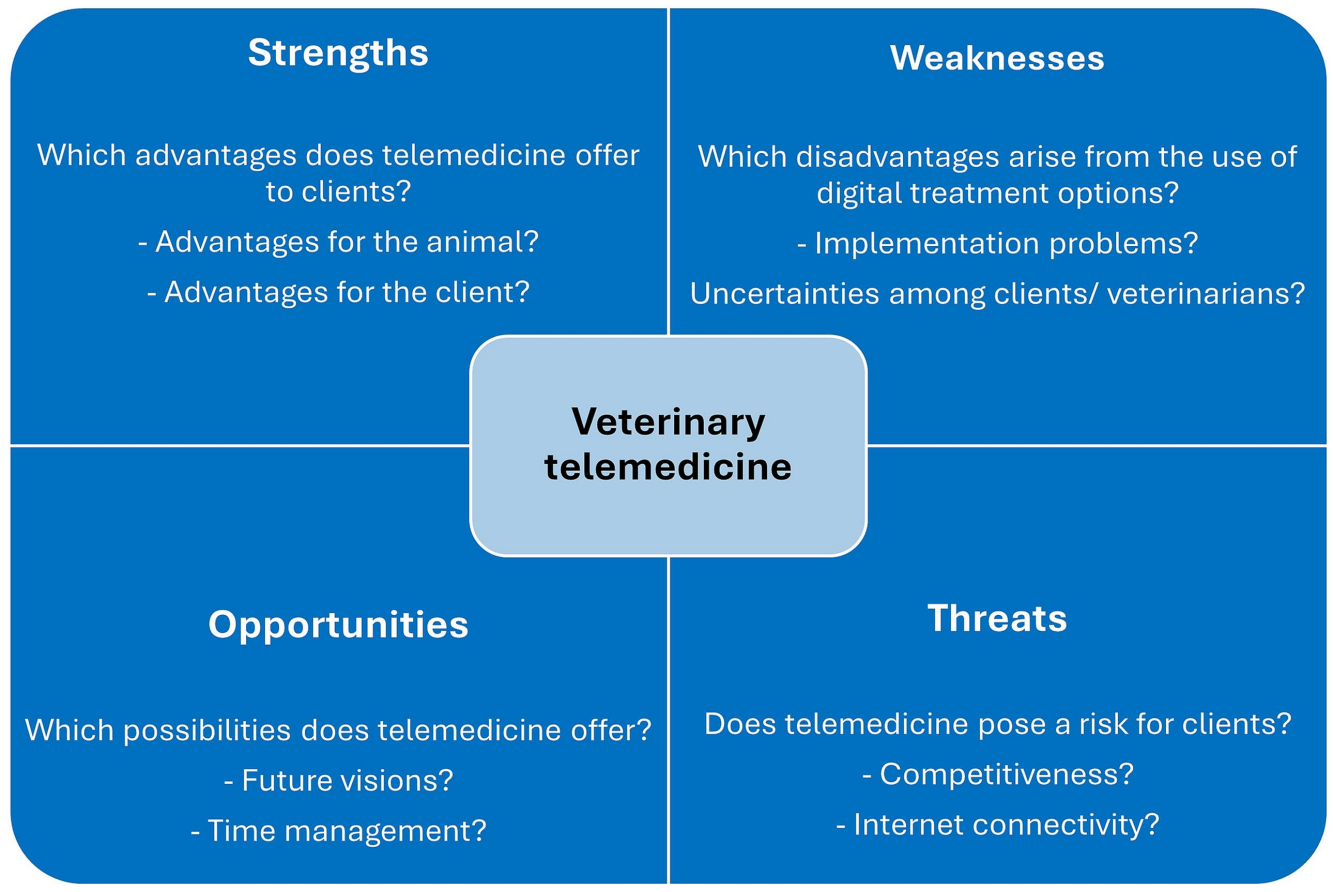
Coding framework for collected quantitative survey responses and qualitative interview data in accordance with SWOT analysis.

To address the research questions, a parallel mixed-method design was performed. This design consisted of quantitative online and paper surveys of small animal owners and qualitative, semi-structured interviews with experts from veterinary practices, veterinary associations, and the pet health insurance industry in Germany. The data were triangulated using descriptive statistics and interpretative analysis. The objective was to methodically compile all collected data within the framework of a SWOT analysis to identify strengths and weaknesses, as well as opportunities and threats in the use of telemedicine for clients (Figure 1).

### 2.1 Quantitative part

The quantitative data were collected using a structured questionnaire based on studies on veterinary telemedicine (20, 21) and adapted to the specific target group. The questionnaire encompassed five distinct areas: (1) sociodemographics and digital infrastructure, (2) previous use and assessment of telemedicine, (3) COVID-19 pandemic-related changes, (4) willingness to pay and value, and (5) evaluation of veterinary telemedicine services. In addition to closed questions, free-text comments were also utilised, which were evaluated using a deductive coding scheme consistent with SWOT analysis. The survey was conducted from April 2023 to April 2024 via the online-survey platform LimeSurvey® and disseminated through social media channels. Furthermore, the questionnaire was distributed in paper form in four small animal practices to reach target groups with less technological literacy. The paper-based responses were also digitalised using LimeSurvey® for analysis. The data from both questionnaire formats were exported and further processed in Microsoft Excel®, where they were descriptively analysed and results were subjected to deductive content analysis (22, 23).

### 2.2 Qualitative part

Furthermore, eight qualitative interviews were conducted with experts, including practicing veterinarians from both urban and rural areas, representatives of a veterinary chamber, the national Association of Practicing Veterinarians (bpt), as well as representatives of pet health insurance companies. To recruit experts for the semi-structured interviews, a purposive and convenience sampling strategy was employed. A list of potential experts was compiled through a combination of expert recommendations, and professional networks. Specifically, we identified 10 experts in the field of veterinary telemedicine who had a minimum of 5 years of experience and a proven track record of research or practice in veterinary telemedicine. These experts were contacted via email or phone, and a brief description of the study was provided to gauge their interest and availability for an interview. A total of eighth experts agreed to participate. The participants for the qualitative interviews were selected based on their professional relevance to the topic of veterinary telemedicine. The sampling focused on individuals who work directly with telemedical services, possess expertise in legal and regulatory frameworks, represent the interests of veterinarians, or are involved in communication processes between veterinarians and pet owners, particularly regarding reimbursement and insurance issues. The inclusion criteria were limited to willingness to participate, regional representation, and knowledge, interest, or practical experience in the field of telemedicine. Sampling was concluded after eight interviews, as no new thematic insights and information emerged, indicating that thematic saturation had been reached. This diverse group of experts was chosen to provide a comprehensive understanding of the research topic from multiple perspectives. The interviews were based on a semi-structured guideline structured along the SWOT dimensions (strengths, weaknesses, opportunities, and threats). The conversations were conducted online via Microsoft Teams®, and were subsequently recorded, transcribed, and analysed using deductive content analysis (22, 23).

### 2.3 Data- and SWOT analysis

For the analysis of the interviews and surveys, a deductive content analysis was conducted (22, 23). Categories were predefined based on the research questions and theoretical considerations, namely the categorisation of content to strengths (S), weaknesses (W), opportunities (O) and threats (T). The coding framework described the four main categories as follows:

- Strengths (S): positive attributes, resources, or capabilities
- Weaknesses (W): limitations, deficiencies, or vulnerabilities
- Opportunities (O): favourable conditions, trends, or possibilities for growth/advantages
- Threats (T): challenges, risks, or negative trends posing potential harm.

Meaningful units of analysis (e.g., sentences, distinct ideas) relevant to the SWOT dimensions were systematically identified within the data and then assigned to these categories (24). The first author ensured that the coded units accurately reflected the operational definitions. Ambiguous content was discussed and resolved in consensus meetings among all authors. Finally, the content within the SWOT categories was analysed considering patterns and salient themes. Relationships and tensions between categories were subjected to strategic interpretation for practical application.

The integration of qualitative and quantitative datasets from online and paper surveys, as well as expert interviews, provides a differentiated basis for evaluating current usage patterns, key challenges and future development prospects (25) of telemedicine in small animal practice. A SWOT analysis was conducted to structure the findings and derive a practical action model. Within the SWOT framework, the qualitative interview findings and the quantitative survey results were systematically synthesised. By assigning both datasets to the same deductively defined categories, the SWOT model functioned as the analytical interface through which the triangulation of results was realised. This step enabled a consolidated interpretation of strengths, weaknesses, opportunities and threats, allowing converging and complementary insights from both methodological strands to be integrated. This approach facilitates a nuanced categorisation of identified factors into the four distinct categories of strengths, weaknesses, opportunities and threats (26). The SWOT analysis has been utilised not only in business and strategic corporate decision-making contexts, but also in scientific settings as a standalone diagnostic instrument. It thus offers a structured approach to visualise the strengths and weaknesses of the status quo, as well as external factors that can act as opportunities or threats (26). The merits of SWOT analysis for interdisciplinary research approaches are also highlighted, as it fosters strategic orientation and provides a catalyst for further development (27). The data utilised in this study were evaluated based on a coding scheme and assigned to the SWOT categories accordingly (Figure 1). Notwithstanding the recognised methodological limitations, including the subjective evaluation of individual factors and the constrained generalisability, the SWOT analysis functions as a constructive instrument for the documentation of salient subjects and the formulation of recommendations for action concerning the integration of digital care services in small animal practice (26).

## 3 Results

The results are presented in different sections to provide a clearer overview, although the collection of survey and interview data was performed in parallel. A comprehensive analysis of the survey results is presented, encompassing both online and paper formats. The analysis includes sociodemographic characteristics, history of telemedicine utilisation, assessment of telemedicine, the impact of the COVID-19 pandemic, the willingness to payment and the perceived value and evaluation of telemedicine services. A summary of the interview results is provided. Finally, the data are categorised based on their quantitative and qualitative properties in relation to the SWOT analysis.

### 3.1 Survey results

A total of 426 questionnaires (online: *n* = 220, paper: *n* = 206) were completed during the survey. All of the 206 surveys filled out in paper format were completed in full and subsequently analysed. Of the 220 online surveys, 198 were fully completed and, subsequent to plausibility checks, were utilised for data collection. Incomplete forms or surveys lacking consent to the privacy policy were excluded from the subsequent analysis, so that 404 responses were included in the analysis.

### 3.2 Sociodemographic characteristics

Similar distribution of responses regarding age, gender, place of residence or distance from veterinarian were received in both formats, whereas more females participated in general and more frequently in the online format (Table 1).

**Table 1:** Sociodemographic characteristics of survey respondents (*n* = 404).

Demographic characteristics	Categories	Absolute values (n)			Relative values (%)		
		Paper	Online	Total	Paper	Online	Total
Age	Max	76	72				
	Min	18	20				
	Mean	47	46				
Gender	Male	79	34	113	38.35%	17.17%	27.97%
	Female	121	159	280	58.74%	80.30%	69.31%
	Diverse	5	0	5	2.45%	0	1.24%
Place of residence	Major city (> 20.000–100.000 inhabitants)	121	76	197	58.74%	38.38%	48.76%
	Medium-sized city (> 5.000–20.000 inhabitants)	35	41	76	17%	20.71%	18.81%
	Small town (< 5.000 inhabitants)	12	19	31	5.83%	9.60%	7.67%
	Rural area	38	62	100	18.45%	31.31%	24.75%
Travel distance to veterinary practice	< 5 km	108	86	194	52.43%	43.43%	48.02%
	> 5–10 km	70	62	132	34%	31.31%	32.67%
	11–25 km	18	29	47	8.74%	14.65%	11.63%
	> 25 km	9	14	23	4.36%	7.07%	5.69%

The majority of survey participants (64.36%, *n* = 260) reported owning a dog, 46.04% (*n* = 186) indicated ownership of a cat, and 12.13% (*n* = 49) stated they had a small mammal. Thirteen respondents (3.22%) selected owning a bird, while 12.38% (*n* = 50) reported owning a reptile. Approximately one third of the respondents stated that they owned more than one pet (*n* = 132, 32.67%). Of these respondents 82.58% (*n* = 109) indicated that they owned two animals, 15.91% (*n* = 21) indicated that they owned three pets, and 1.52% (*n* = 2) indicated that they had four or more pets. A descriptive comparison of the paper and online versions of the survey indicated no notable differences in the distribution of pet species (Table 2).

**Table 2:** Proportion of pet species by survey method (*n* = 404), multiple responses possible.

Species	Survey method	Number of responses (n)	Percentage of responses (%)
Dogs	Paper survey	129	31.93%
	Online survey	131	32.43%
	Total	260	64.36%
Cats	Paper survey	102	25.25%
	Online	84	20.79%
	Total	186	46.04%
Small mammals	Paper survey	24	5.94%
	Online survey	25	6.19%
	Total	49	12.13%
Birds	Paper survey	7	1.73%
	Online survey	6	1.49%
	Total	13	3.23%
Reptiles	Paper survey	31	7.67%
	Online survey	19	4.70%
	Total	50	12.38%

### 3.3 Telemedicine usage, options and current knowledge

**Figure 2:**
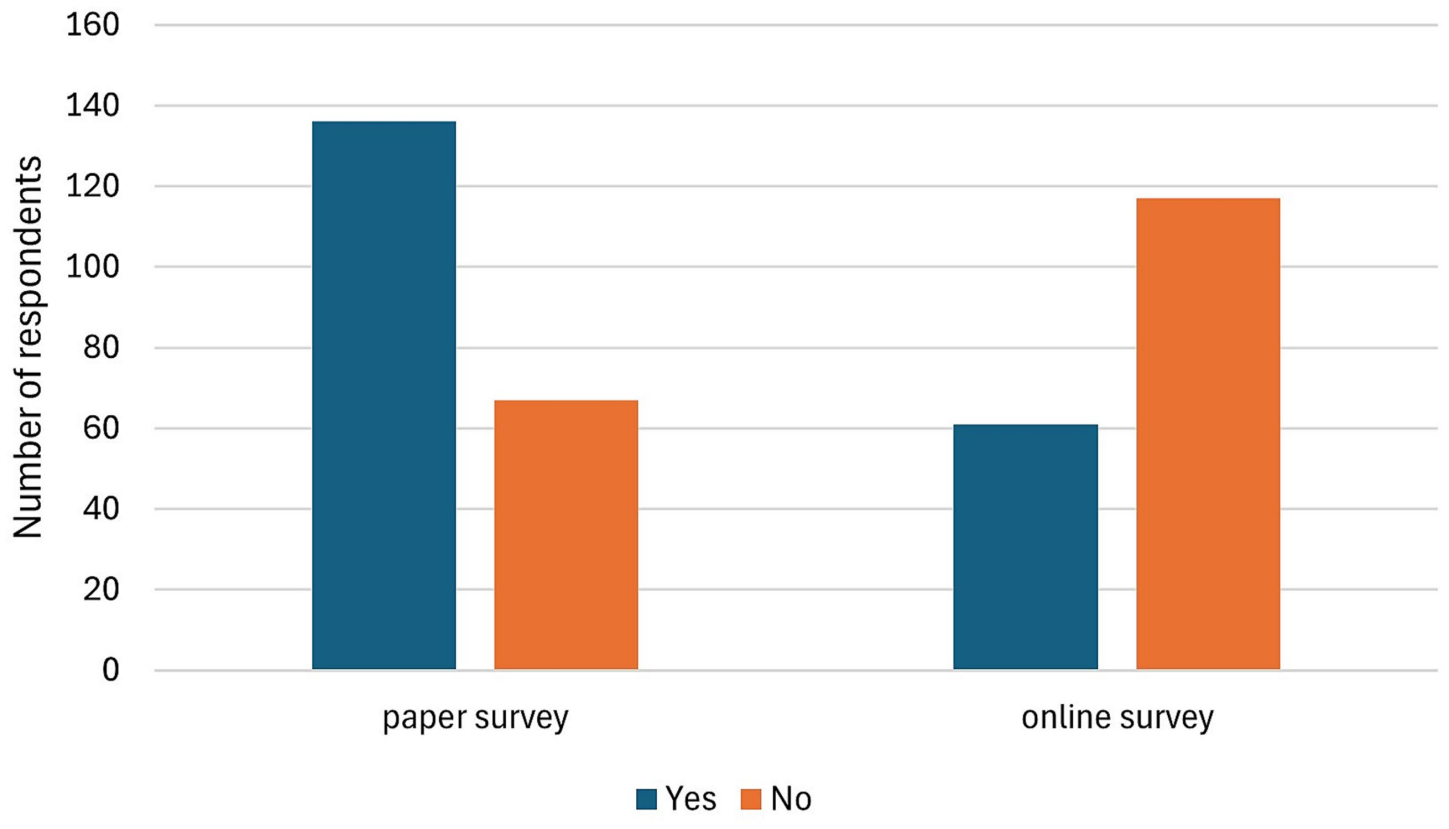
A comparison of both survey formats (paper and online survey) for the use of digital services by animal owners for contacting the veterinary small animal practice (*n* = 404).

The survey revealed that 61.63% of the participants were acquainted with the term “telemedicine,” while 136 individuals denied familiarity with it. In the paper survey, 58.74% of respondents confirmed their knowledge; in the online survey, this proportion was higher with 64.65%. The participants were also asked whether they had previously utilised digital media for the purpose of contacting their practice, with the exception of scheduling appointments. The survey revealed that 48.76% of respondents answered in the affirmative, indicating their familiarity with digital means of contact. Conversely, 45.54% of respondents indicated that they had not yet adopted such technologies. A further 5.69% of respondents did not provide an answer. A subsequent analysis of the subgroups indicated that only 30% of online respondents had utilised this option, while 66% of paper survey respondents said they had (Figure 2).

**Figure 3:**
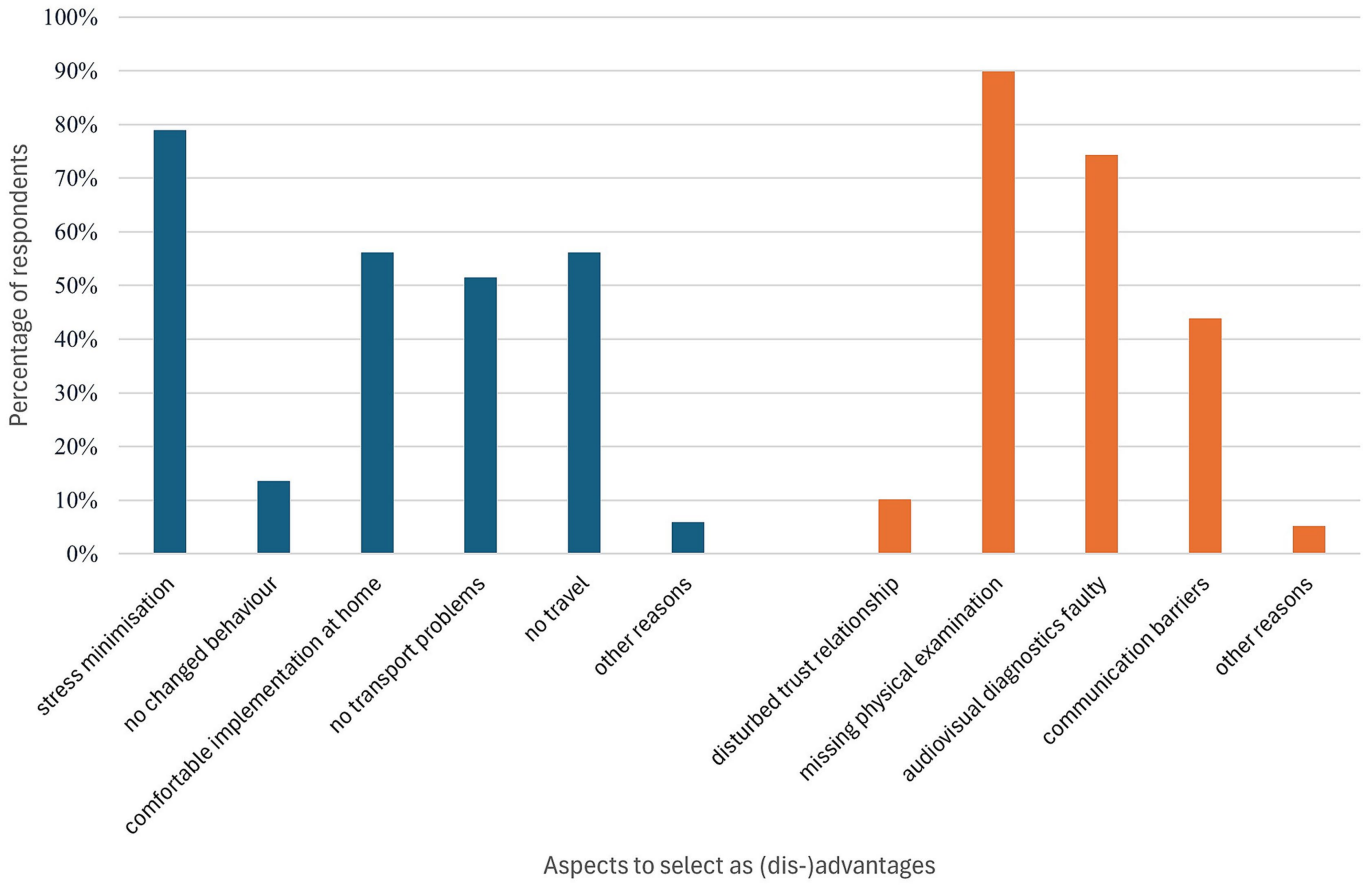
Client perspectives on the benefits (blue) and disadvantages (orange) of telemedicine utilisation (*n* = 404; multiple selection was possible).

Digital communication channels were primarily utilised for the discussion of findings (84.26%, *n* = 166) and the clarification of ongoing treatment issues (85.79%, *n* = 169). The scope of application in other areas, such as the provision of feeding or husbandry advice (*n* = 50), was comparatively limited with 25%. Other areas of digital contact occured regarding questions on animal husbandry or handling, appointment or billing issues. The primary benefits of telemedicine were identified as the mitigation of stress for the animal, the convenience of conducting the procedure from a domestic setting, and the elimination of transportation requirements. The minimisation of stress was cited by 319 respondents. Only 13.61% of small animal owners selected changes in behaviour during the examination in practice rooms, and thus a possible absence of abnormal signs. A total of 227 subjects indicated that the convenience of performing the procedure in the home environment was a beneficial aspect of the study. Meanwhile, 208 participants expressed satisfaction with the elimination of transportation-related challenges. Furthermore, additional aspects such as accelerated performance, the future potential of digital solutions, and improved accessibility were highlighted in a few free-text responses (*n* = 23). With respect to potential disadvantages, the absence of a physical examination was identified as a significant point of criticism by 89.85% of respondents. The audiovisual modality of diagnostics was also met with critical scrutiny: three hundred individuals expressed apprehension regarding the inadequate dissemination of information and the concomitant possibility of misinterpretation. A total of 177 participants indicated that the absence of in-person consultation had the potential to result in communication difficulties. In contrast, only a small proportion of respondents (*n* = 41) perceived the relationship of trust between veterinarians and pet owners as being compromised by telemedicine. A total of 21 respondents expressed concerns regarding negative personal experiences with digital services, technical challenges, and access barriers for users with low digital affinity. These concerns were addressed in free-text contributions. The advantages and disadvantages from small animal owners’ perspectives are presented in Figure 3.

### 3.4 Impact of the COVID-19 pandemic on telemedicine use

The impact of the COVID-19 pandemic on attitudes towards the utilisation of telemedicine was found to be negligible. The majority of respondents (*n* = 295) indicated that their stance on telemedicine remained unaltered in the wake of the pandemic. Among the participants who reported a change in their attitudes (*n* = 40), the comments generally reflected a more positive outlook. The experiences that individuals encountered during the pandemic resulted in a notable increase in receptivity to digital healthcare services. This increased receptivity was characterised by an emphasis on the benefits that these services offer, such as the potential for time savings and improved accessibility.

### 3.5 Value and willingness to pay in veterinary telemedicine

**Figure 4:**
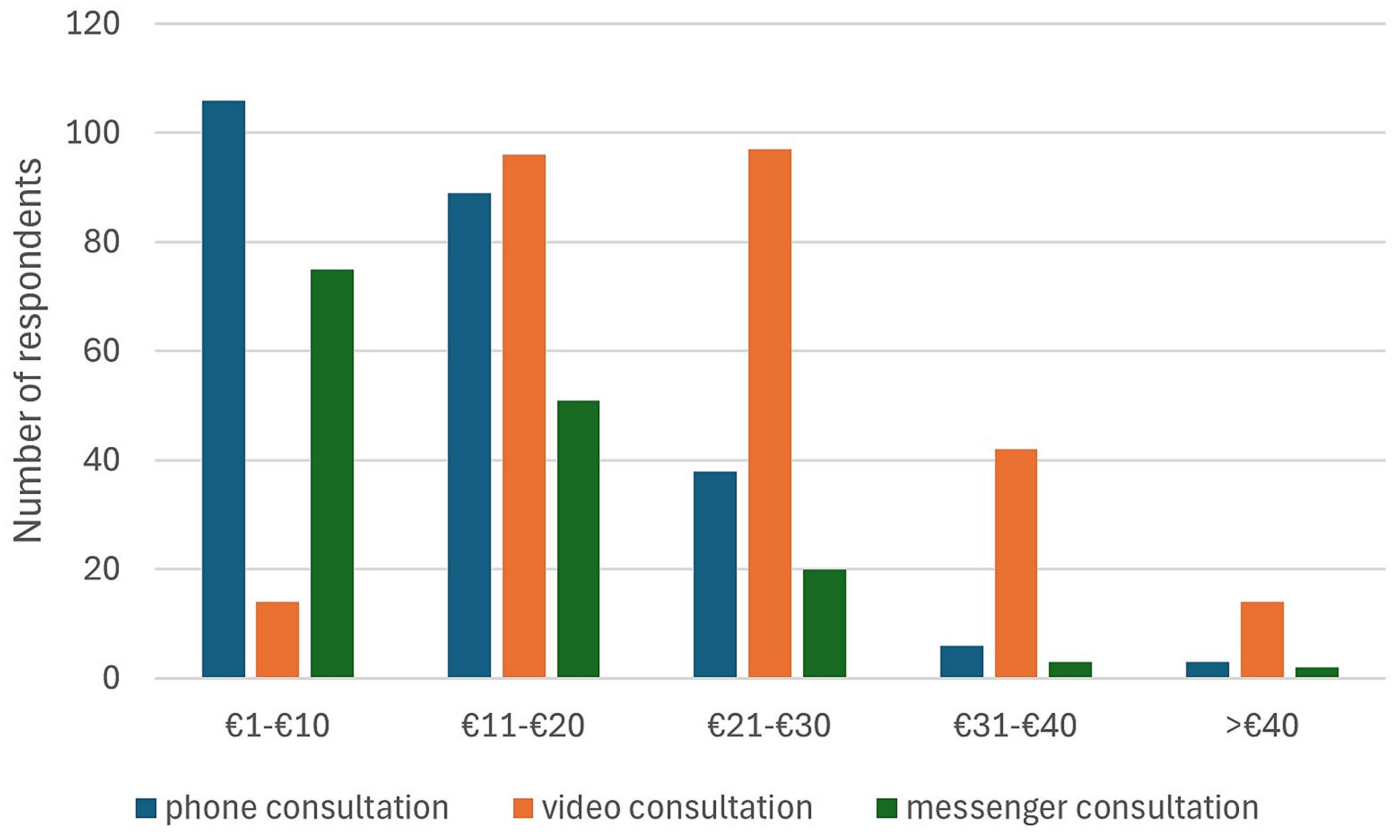
Distribution of customer opinions on the willingness to pay for digital veterinary services (*n* = 286; multiple selection possible).

The preliminary assessment of willingness to pay (WTP) was designed to provide an initial descriptive overview rather than a validated economic measure. The items were piloted with a small group (*n* = 8) to ensure clarity and comprehensibility. WTP was collected through categorical response options, with defined boundaries (Figure 4). As the measure consisted of single categorical items, internal reliability indices are not applicable. Potential influences of social desirability were considered when interpreting the results; therefore, only descriptive analysis was conducted. The results showed a generally high willingness to pay for telemedicine services among respondents, particularly for audiovisual formats, such as video consultations, with 93.36% of participants being willing to pay. Remuneration expectations varied considerably depending on the communication format. Video consultations were rated highest in terms of both acceptance and WTP, with many respondents placing them in a medium price category. For telephone consultations, which 67.13% considered worthy of payment, a lower fee tended to be considered appropriate. WTP appeared more reserved for text-based formats such as email or messenger communication. Sixty-two participants perceived this as a paid service for which a lower fee was more commonly considered appropriate. For a messenger chat with veterinarians, 93 respondents stated that they would be willing to pay for this telemedicine service. Overall, the assessment of the value of telemedicine services depended on the type of consultation (Figure 4).

**Figure 5:**
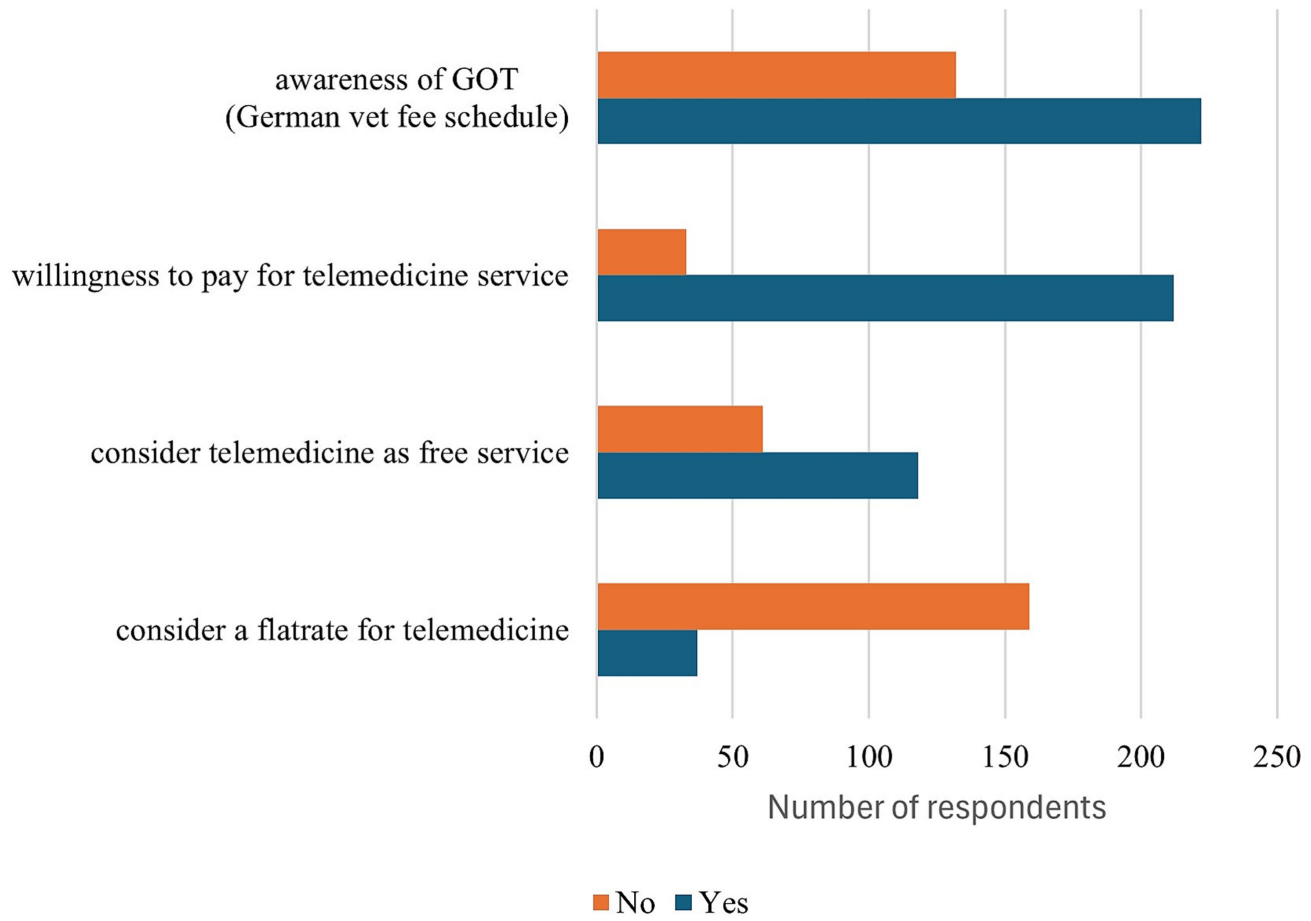
Overview of responses to survey questions relating to flat-rate usage, willingness to pay, and awareness of the German veterinary fee schedule (GOT; *n* = 404).

The survey results show a predominantly cautious attitude towards the implementation of a flat-rate model for audiovisual consultations. The data indicate that 55.59% of the participants expressed opposition to the proposed flat-rate solution, while 12.94% showed their support for this option. An additional 29.72% of respondents indicated that they could potentially envision a flat rate. The general classification of telemedicine services also presents a differentiated picture: while a majority of respondents (56.38%) recognised this as a fee-based service, 31.38% perceived it as being more of a free service. It is noteworthy that 12.23% of the respondents did not provide an answer. The inquiry concerning the awareness of the German veterinary fee schedule (GOT) disclosed that 59.04% of small animal proprietors are aware of this. Conversely, 35.12% of respondents expressed a negative stance, while 5.85% did not answer (Figure 5).

**Figure 6:**
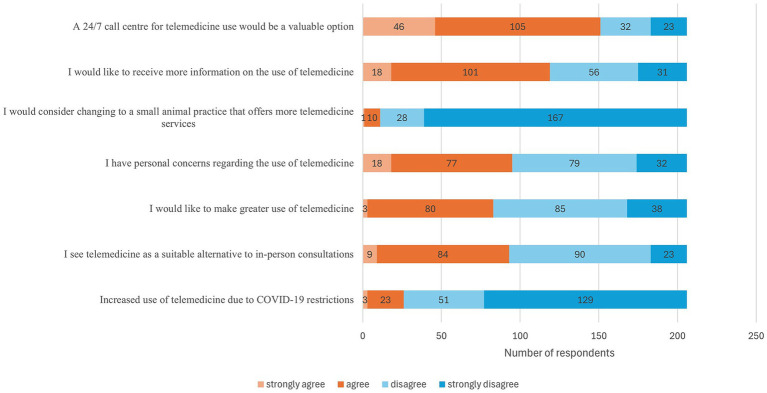
Client evaluation questions on the use and possibilities of telemedicine, paper format (*n* = 206).

**Figure 7:**
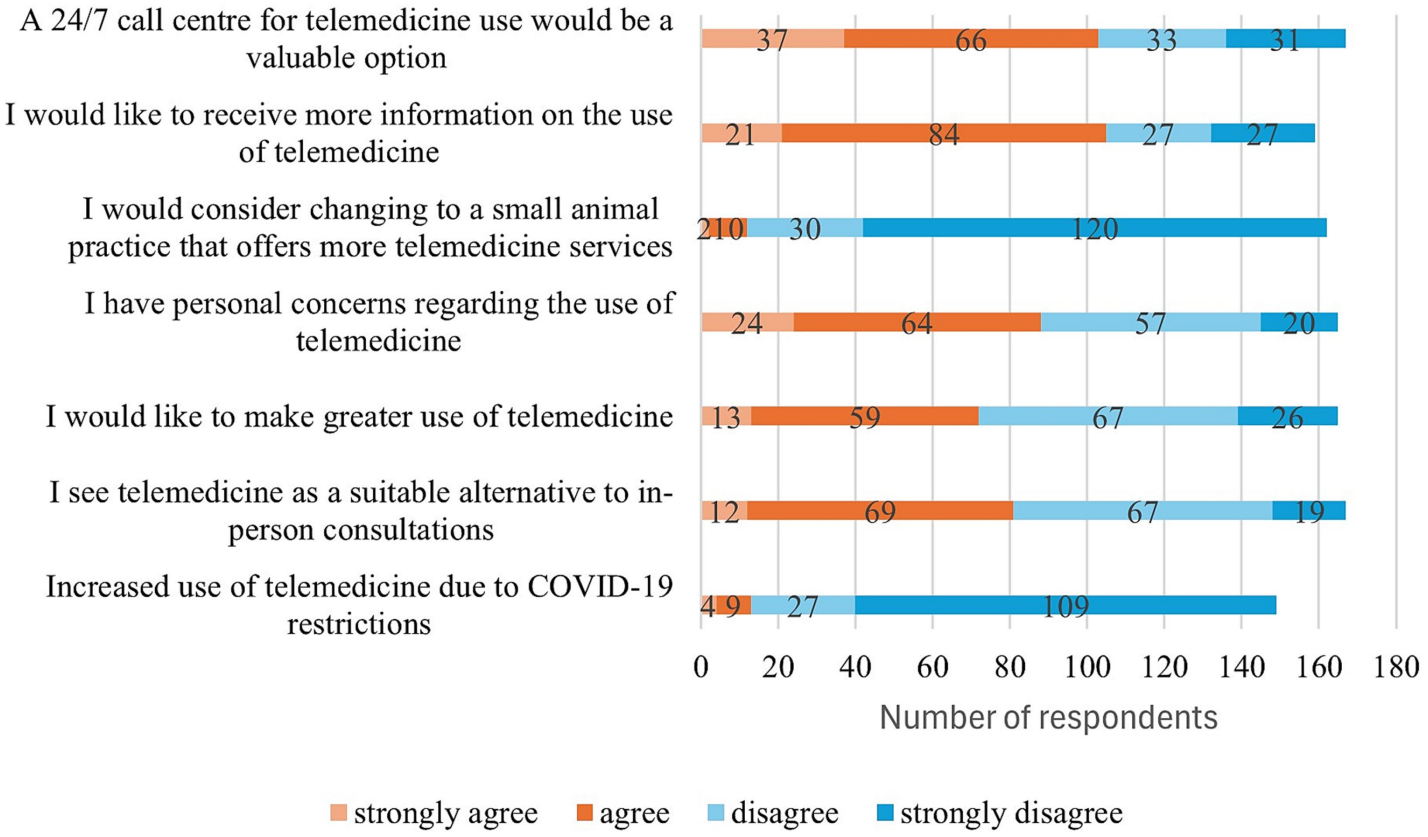
Client evaluation questions on the use and possibilities of telemedicine, online format (*n* = 198).

The final section of the client questionnaire was designed to explore attitudes towards telemedicine services and the acceptance of audiovisual consultations. It contained various evaluation statements. The majority of respondents indicated that the restrictions imposed by the pandemic had not led to an increase in their use of telemedicine consultations. A few respondents reported an increase or newfound interest in digital services due to the contact restrictions. There was a divergence of opinions regarding the potential of telemedicine to serve as a viable substitute for in-person consultations. The reception of digital consultations was varied, with some respondents considering them innovative or supportive additions to existing practices, while others did not regarded them as equivalent substitutes. Notably, some individuals explicitly rejected digital consultations, highlighting the diversity in attitudes towards this emerging form of communication in healthcare. The inclination towards a more pronounced utilisation of telemedicine services was met with a tempered response. While a proportion of respondents expressed interest, a predominant sentiment of scepticism or a sense of distance prevailed. Approximately one-third of the participants in the study expressed reservations regarding the use of telemedicine. Conversely, a more substantial group exhibited no discernible reservations or only exhibited nominal reservations, suggesting a range of experiences and levels of confidence in digital formats. However, only a small percentage of pet owners would consider changing their current veterinary practice to enhance access to telemedicine services. For the majority of respondents, this was not a salient factor for choosing the veterinarian. Approximately half of the participants expressed a desire for further information regarding telemedicine and its potential applications. Concurrently, a considerable proportion expressed satisfaction with the prevailing state of knowledge or perceived no necessity for further information. A 24-h veterinary consultation service, for example, via a call centre, was met with approval by many. The notion of designated contact personnel who are consistently accessible was regarded as reasonable by a substantial proportion of the participants, although a considerable number exhibited a degree of scepticism regarding this concept (Figures 6, 7).

### 3.6 Interview results

As part of the study, semi-structured interviews were conducted with representatives from veterinary practices, veterinary associations, professional associations, and pet health insurance companies. The objective of the interviews was to contextualise practical experiences and expert assessments in relation to the results of the pet owner survey. In total, eight experts agreed to participate in the interview, which represents a response rate of 80% of invited experts. The duration of the interviews ranged from a minimum of 45 min to a maximum of 90 min (mean 67.5 min). Data saturation was achieved, on the one hand, by covering the targeted and convenient sampling strategy, and on the other hand, the interviewer noticed that no new topics or aspects cropped up, there being a repetition of responses regarding topics and ideas and a decrease in the amount of new information collected. The experts unanimously highlighted significant time savings and flexibility that telemedicine offers.

One veterinarian commented: *“Digital consultations often serve as a first point of contact or a kind of ‘first aid’ for our patients.”*

Digital consultations can also help to prioritise cases according to urgency and optimise practice workflows: *“Telemedicine is particularly beneficial for people with limited mobility or for specialities where specialised knowledge needs to be disseminated quickly.*”

Obstacles were identified, including technical issues such as unstable internet connections, inadequate equipment and limited digital literacy. As one expert explained: *“The heterogenous infrastructure leads to very difficult usage options, and unclear legal regulations and billing modalities complicate practical implementation.”*

In addition, the proliferation of incompatible software solutions was mentioned as a factor contributing to organisational challenges and competitive pressure. The experts also discussed the future potential of telemedicine: *“I see opportunities mainly in relieving emergency services, improving networking between veterinarians, specialists, and pet insurance companies, and simplifying documentation and billing.”*

Another interviewee added: *“Low-threshold access could be particularly attractive to digitally savvy generations, and AI could help standardise diagnoses and minimize errors.”*

On the other hand, other experts pointed out risks associated with the use of AI: *“There is a risk of misdiagnoses by inexperienced veterinarians, and investment and training costs should not be underestimated. In addition, customer loyalty could suffer if telemedicine is not used appropriately.”*

In practice, telemedicine has proven particularly useful for follow-up care, wound checks, feeding advice and behavioural abnormalities, as animals can be evaluated in their familiar environment. As one veterinarian emphasised: “*As long as telemedicine is used as a supplement to in-person consultations, the trust relationship with our regular clients is not compromised.”*

According to experts in the field, the pandemic served as a catalyst for acceptance, though it was not the sole cause of this development. Health insurance companies have already established comprehensive reimbursement policies for telemedicine services within partner networks. However, uncertainties persist in external contexts. The maintenance of uniform standards, targeted training, and improved integration into the fee schedule are considered essential for long-term success.

### 3.7 Results of SWOT analysis

**Figure 8:**
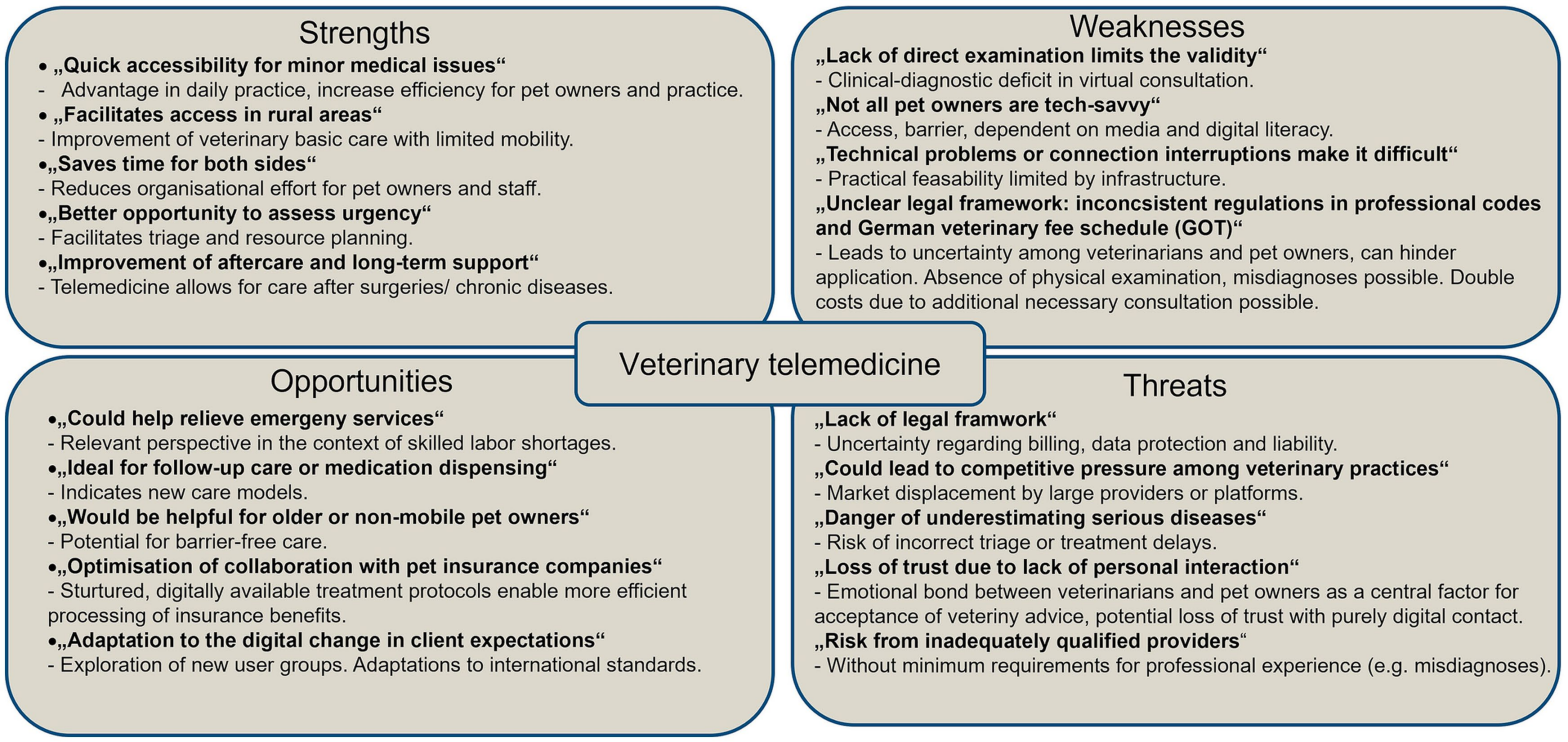
SWOT analysis of telemedicine use based on client and expert opinions.

The evaluation of telemedicine by pet owners and experts reveals a nuanced perspective, underscoring both its potential benefits and the challenges it faces (Figure 8).

#### 3.7.1 Strengths

The following list outlines the strengths of this topic: The practicality of telemedicine is emphasised by both clients and experts. The advantages of this approach are manifold, including, but not limited to remarkable time savings, enhanced flexibility, and the uninterrupted availability of veterinary advice, irrespective of one’s geographical location. Pet owners have noted the convenience of being able to address minor issues or inquiries from the comfort of their own homes, eliminating the need for stressful visits to the veterinary practice or extended waiting times. Furthermore, experts underscore the structural advantages for practice organisation. Telemedicine has the potential to facilitate streamlined appointment processes and expedite the assessment of urgent issues. Digital consultations have been particularly beneficial for individuals with limited mobility, those residing in rural areas, and those with physical impairments. Experts have identified additional benefits of telemedicine, including its potential in specialised fields such as behavioural science and the enhanced accessibility of veterinary specialists.

#### 3.7.2 Weaknesses

Notwithstanding the aforementioned advantages, concerns have been voiced by both parties. Many pet owners have expressed concerns regarding the reliability of diagnostic procedures that do not involve a physical examination of their pet. The erosion of the personal connection between veterinarians and pet owners is also viewed critically. The presence of technical challenges, including, but not limited to unstable internet connections, inadequate equipment, and a lack of digital skills especially among older users, has been shown to have an additional inhibitory effect. Moreover, experts have identified organisational challenges. The GOT is not yet consistently geared to telemedicine services, which leads to billing uncertainties. The proliferation of platforms and software solutions engenders notable challenges in terms of orientation, both for veterinary practices and their clients. Moreover, the legal framework is frequently inconsistent, leading to legal uncertainty. Language barriers and misunderstandings are also identified as potential obstacles to digital communication.

#### 3.7.3 Opportunities

Telemedicine has been shown to engender a broad spectrum of development opportunities. It has been demonstrated that clients perceive considerable promise in the implementation of AI, particularly with regard to the standardisation of diagnoses and the mitigation of errors. Experts further emphasise the opportunity to reach new target groups, such as tech-savvy pet owners or people with limited access to veterinary care, and to address existing gaps in care. Furthermore, they regard telemedicine as a valuable instrument for alleviating the workload of existing emergency services. This can be achieved through the implementation of digital triage systems. The increasing popularity of telemedicine platforms, a generation shift in user behaviour (“mobile-first”), and the desire of many pet owners for better information are promoting acceptance. Moreover, digital documentation and treatment protocols have the potential to enhance transparency and traceability for pet owners.

#### 3.7.4 Threats

However, this potential for growth is tempered by substantial threats or risks that could impede the broader adoption of telemedicine. Clients often harbour scepticism regarding the quality, security and data protection of digital services. Concerns regarding security breaches, data loss, and the potential financial implications of duplicated costs (e.g., the necessity of an on-site examination subsequent to an online consultation) impede the acceptance of this approach. Furthermore, experts fear that the requisite “digital restructuring” represents a substantial economic encumbrance, particularly for smaller or rural practices. The dearth of pervasive internet access further exacerbates this predicament, potentially resulting in an inequitable distribution of care prospects in the long term. These challenges are further compounded by the potential for tension to arise in the context of the day-to-day work in veterinary practices. The impact of digitalisation on the veterinary profession has the potential to engender internal strife, particularly among experienced professionals and long-serving employees. Finally, legal uncertainties remain, as evidenced by the potential inconsistency between professional regulations and certain digital treatment methods (Figure 8).

### 3.8 Strategic interpretation of SWOT analysis

The SWOT analysis demonstrates that the successful establishment of telemedicine in small animal practice requires an integrated strategic approach. Strengthening user acceptance through increased trust in digital services and ensuring ease of use plays an overarching role. To this end, telemedical services must be communicated clearly, with their concrete benefits, such as time savings, flexibility, and reduced stress, being highlighted. At the same time, reducing uncertainties regarding diagnostic reliability, data protection, and potential additional costs is essential, as these factors strongly influence users’ willingness to adopt such services. From an organisational perspective, telemedicine requires greater professionalisation and standardisation of practice workflows. Consistent documentation processes, reliable software solutions and clear billing and legal frameworks provide orientation and reduce both internal and external barriers. Sustainable implementation additionally requires target-group-specific support measures that take different levels of digital competence into account. Training initiatives to strengthen internal acceptance as well as transparent communication with pet owners are essential prerequisites for generating positive user experiences. Investments in stable internet connections, appropriate hardware and continuous competence development represent key strategic elements. Overall, the findings suggest that telemedicine can be implemented successfully particularly when it is understood as a complementary, quality-enhancing service that improves veterinary care while simultaneously addressing the needs of its users.

## 4 Discussion

Telemedicine is gaining importance in veterinary medicine, and in the wake of advancing digitalisation, it is regarded as a future-oriented complement to traditional consultations (28). While previous studies have focused on the perspective of veterinarians (20), the present study deliberately focuses on the perspective of pet owners. The findings indicate that owners’ expectations, concerns and usage habits play a pivotal role in the successful implementation of telemedicine applications. With regard to the research conducted by Becker (20), who examined the perspectives of veterinarians on telemedicine, the present study shows that similar thematic areas can also be identified on the client side. In particular, the digital competence of animal owners, their expectations regarding the usefulness and quality of telemedical services and the infrastructural conditions (e.g., internet access, technical equipment, data protection) emerge as key factors influencing the acceptance and use of telemedicine. These findings indicate that the implementation of telemedicine in veterinary medicine represents not only a technological but also a socio-cultural and structural challenge affecting both veterinary professionals and animal owners. Consequently, it is imperative to investigate and document the needs, experiences and reservations and to take these into account in the ongoing development of digital care offerings. The observed acceptance and perceived usefulness of veterinary telemedicine among pet owners can be interpreted through the lens of the TAM (3) and the UTAUT (4). According to these frameworks, factors such as perceived usefulness, ease of use, trust in the veterinarian and facilitating conditions help explain participants’ attitudes and intentions towards telemedical services. While TAM and UTAUT were not empirically tested in this study, they provide a useful conceptual framework to interpret the results and highlight potential determinants of telemedicine adoption that could inform future implementation strategies and practice recommendations.

The survey indicates that a large proportion of pet owners possess a fundamental understanding of telemedicine. Digital communication channels such as email and telephone are already widely used, particularly for discussing findings or for questions about treatment. Audiovisual formats, including video consultations, have seen only limited adoption to date. However, there is a general openness to their increased use, especially when concrete benefits for pets (e.g., stress avoidance) and time or cost savings for the owners are evident (29). The disparities among animal species give rise to varied assessments. Respondents in this study revealed that owners of small mammals and reptiles exhibited a higher degree of acceptance of telemedicine services compared to owners of dogs and cats. This observation is consistent with the findings of international studies that indicate a higher propensity to utilise digital health applications among less prevalent pet species, as specialised care is often inaccessible (30). For keepers of exotic animals, especially reptiles, spatial distance to specialised clinics, limited transport options and the low density of veterinary service providers constitute structural barriers (30). Under these conditions, telemedicine can take on a central role, as video-based initial assessments, follow-up observations, or triage consultations can facilitate access to expert advice and reduce stress for the animals. At the same time, however, systemic challenges limit the effectiveness of telemedical services for exotic species: studies show clear deficits in the education and training of veterinarians in reptile and small-mammal medicine, resulting in a low number of truly experienced specialists (31). As telemedical decisions rely heavily on specific expertise, differentiated clinical knowledge and the ability to make accurate image-based assessments, insufficient specialisation can substantially impair diagnostic quality and thus the potential of telemedical care. Overall, telemedicine therefore offers an important opportunity to reduce existing inequalities in access for reptile keepers, but at the same time requires sufficient specialisation and continuing education of veterinary personnel to ensure high-quality remote care.

From the respondents’ perspective, the advantages of telemedicine services primarily lie in increased flexibility, time savings and reduced stress for the animal. A particularly salient finding is that 79% of respondents identified minimising stress for the animal as their most outstanding benefit. This finding aligns with the conclusions of other studies which identified stress reduction as a pivotal component of pet healthcare (29). On-site consultations have been demonstrated to result in quantifiable physiological stress reactions (32) despite the continuing emphasis on stress-reduction and low-trigger processes (33). Aspects of daily organisation play a central role in this matter. According to our findings, 56% of respondents perceived the convenience of performing the procedure at home as an advantage. Furthermore, 51% of respondents emphasised the elimination of transportation problems. Concurrently, however, numerous reservations were also expressed: the absence of a physical examination, the ambiguities surrounding diagnosis, and the absence of emotional connection due to digital formats were frequently mentioned as disadvantages. A high proportion of the participants expressed concerns regarding the absence of a physical examination, with approximately 90% citing it as a notable drawback.

Telemedicine consultations may increase the risk of misdiagnosis when essential clinical information is missing or when symptoms cannot be reliably assessed via video (34). In addition, there is a risk of delaying necessary in-person examinations, particularly when owners underestimate the severity of a problem or when technical barriers impair communication. The quality of clinical assessment depends heavily on the information provided by owners, which may be subjective, incomplete or inaccurate. The Five Freedoms framework provides a suitable reference point for systematically assessing animal welfare-related aspects (35). Telemedicine must not compromise any of the Five Freedoms, particularly freedom from pain, injury and disease, nor freedom from fear and distress. Instead, telemedicine should be designed to enable early consultation, minimise risks and improve access to veterinary expertise without compromising the quality of care. Although stress reduction for animals has been identified as a potential benefit of telemedicine, it is equally important to consider possible risks to animal welfare. A central concern is the risk of misdiagnosis or delayed diagnosis when a physical examination is limited or not possible. Telemedical consultations rely heavily on the information provided by animal owners, such as descriptions, videos or photos. The quality, completeness, and accuracy of these materials can vary greatly, which may limit veterinarians’ ability to adequately assess the animal’s condition. If relevant symptoms are unintentionally omitted or clinical signs are misinterpreted, this can lead to insufficient or delayed interventions. Furthermore, ethical challenges may arise when telemedicine is used as a substitute rather than a complement to in-person visits, especially when financial constraints prevent owners from seeking timely veterinary care. In such cases, telemedicine may inadvertently exacerbate existing inequalities in access to adequate diagnostics and treatment. This raises the question under which circumstances animals may not receive the necessary care to safeguard their welfare and comply with the Five Freedoms framework (36). Additionally, 74% of the respondents voiced disapproval of diagnostics that were exclusively audiovisual in nature. The acceptance of technology is often hindered by various technical challenges, including unstable internet connectivity and inadequate device infrastructure (37). Data protection and trust in the digital infrastructure or security also play a key role. The potential for misdiagnosis due to the provision of services by inadequately qualified providers and the absence of legal regulations has been underscored by both pet owners and experts in alignment with international evaluation (38, 39).

A key finding of our study is the pronounced demand among pet owners for information regarding pet care. In both survey formats, 50% of respondents indicated that they expressed a desire for further information regarding the potential applications, legal framework and cost structures associated with telemedicine. The prevailing perspective among the majority of respondents was that telemedicine is not regarded as a substitute for traditional consultations; rather, it is considered a complementary option. Nevertheless, the respondents expressed a general interest in expanding these services, as was the case in previous studies (40, 41). The results clearly demonstrate that the successful establishment of telemedicine services in veterinary medicine does not depend solely on technological innovations. Rather, it requires clear legal requirements, more uniform billing models, and trust-building measures. Veterinary professional associations, universities and veterinary chambers must play a key role and develop both information services, training and continuing education formats for veterinary practices (42). Moreover, it is imperative for pet owners to play a more active role in the development of these services. This approach is essential for identifying solutions that align with the actual needs of the population. The integration of personal care services with supplementary digital offerings, collectively referred to as “hybrid care models,” is poised to emerge as a leading paradigm for the future of healthcare (43). The Five Freedoms framework provides a suitable reference point for systematically assessing animal welfare-related aspects. Telemedicine must not compromise any of the Five Freedoms, particularly freedom from pain, injury and disease as well as freedom from fear and distress. Instead, telemedicine should be designed to enable early consultation, minimise risks and improve access to veterinary expertise without compromising the quality of care. In a European comparison, it becomes clear that the regulation of veterinary telemedicine varies considerably between countries. In Germany, no uniform statutory framework exists to date; instead, the existing guidelines are based primarily on professional recommendations and position papers issued by the BTK and the regional veterinary chambers. In contrast, the United Kingdom has established clearer regulations. The Royal College of Veterinary Surgeons (44) developed a comprehensive professional code that includes specific guidelines for telemedicine. Remote consultations and follow-up care are clearly defined, and recent reforms even allow prescriptions to be issued without a prior physical examination under certain conditions. Although physical examinations remain the recommended standard, the available recommendations provide a high degree of legal certainty. In practice, this results in a comparatively liberal and well-structured implementation of telemedical services. Austria also has more formalised regulations than Germany. The Austrian Veterinary Chamber introduced a binding directive on veterinary telemedicine that sets out concrete requirements for consent, documentation, quality assurance and permissible areas of application (45). Telemedicine is explicitly defined as a complementary service whose use is tied to clear standards of diligence and professional competence. In addition, the Federation of Veterinarians of Europe (46) published a pan-European position paper outlining general recommendations for the responsible use of telemedicine. The FVE particularly emphasises the importance of an established veterinary-client-patient relationship (VCPR), clear documentation obligations, and the role of telemedical services as a supplement rather than a substitute for physical clinical examinations (46). Although these recommendations are not legally binding, they serve as an important reference point for many national veterinary chambers and highlight the need for harmonised minimum standards across Europe.

This study is subject to several limitations. First, the survey does not provide nationwide regional coverage, which restricts the generalisability of the findings to the entire Federal Republic of Germany. Furthermore, only a very limited sample size was achieved, when acknowledging that more than 30 million pets were kept in Germany in 2024 (47). Additionally, the convenient survey methods more likely reached participants who expressed stronger support for telemedicine; this could lead to a distorted representation of overall acceptance within our sample. Second, there is a lack of comparative studies examining telemedicine from the perspective of pet owners, which makes contextualisation more challenging. Additionally, the study’s sample population comprised pet owners whose previous experience with digital services exhibited significant variability, a factor that could have introduced bias into the assessments. Furthermore, the engagement with digital health services during and in the aftermath of the pandemic may have influenced the outcomes (48). From the perspective of many pet owners, telemedicine represents an intriguing, albeit not yet fully established, complement to traditional veterinary care. The highest levels of approval are observed for audiovisual formats in face-to-face interactions with a familiar veterinary practice. The long-term acceptance of these measures is contingent upon the provision of transparent information, the establishment of trust in service providers, and the integration of these measures into a hybrid care concept (49). A potential limitation of the assessment of WTP lies in the influence of social desirability bias (50). Respondents may provide answers they perceive as more socially acceptable or appropriate within the research context, which can distort the reported WTP. Especially for topics that carry normative expectations, such as the valuation of veterinary services, it is possible that participants overestimate or underestimate their true willingness to pay. This potential bias should therefore be taken into account when interpreting the findings.

The findings indicate the necessity for future research to employ larger more diverse samples and adopt a more nuanced approach, taking into account differences in animal species, age groups, and geographical locations. Only through additional empirical analyses can we expect to gain long-term insights into acceptance, likelihood of use and willingness to pay. This can make a key contribution to a well-founded, practice-oriented further development of veterinary telemedicine (51).

## Data Availability

The raw data supporting the conclusions of this article will be made available by the authors, without undue reservation.
